# 

**Published:** 1878-06

**Authors:** 


CONTENTS.

| | Page | | Page |
|---|---|---|---|
| Spirometrology . . . . . . | 489 | Our Children's Teachers . | 504 |
| Mathematical Measurement | 491 | Households Contrasted . . | 505 |
| A Weighty Consideration . | 493 | Hair Washes . . . . . . . | 507 |
| Suspiciousness . . . . . . | 495 | Pouting . . . . . . . . . . | 508 |
| How to Sit . . . . . . . . | 495 | Real Corn Bread . . . . . | 510 |
| American Manners . . . . . | 496 | Softening of the Brain . . | 511 |
| How to get up Early . . . | 496 | Dieting for Health . . . . | 512 |
| The American Climate . . . | 497 | Nursing Animosities . . . . | 513 |
| Tobacco: Its Use and End . | 499 | Suggestive . . . . . . . . | 513 |
| The Aim of Life . . . . . | 502 | Finger Nails . . . . . . . | 513 |

